# Neuroprotective Effects of the Pannexin-1 Channel Inhibitor: Probenecid on Spinal Cord Injury in Rats

**DOI:** 10.3389/fnmol.2022.848185

**Published:** 2022-05-19

**Authors:** Qi Qi, Xiao-Xuan Wang, Jing-Lu Li, Yu-Qing Chen, Jian-Rong Chang, Jin Xi, He-Zuo Lü, Yu-Xin Zhang

**Affiliations:** ^1^Clinical Laboratory, The First Affiliated Hospital of Bengbu Medical College, Bengbu, China; ^2^Anhui Key Laboratory of Tissue Transplantation, Bengbu Medical College, Bengbu, China; ^3^School of Laboratory Medicine, Bengbu Medical College, Bengbu, China; ^4^School of Basic Medicine, Bengbu Medical College, Bengbu, China

**Keywords:** probenecid, spinal cord injury, inflammasome, macrophages polarization, immune microenvironment

## Abstract

Proinflammatory immune cell subsets constitute the majority in the local microenvironment after spinal cord injury (SCI), leading to secondary pathological injury. Previous studies have demonstrated that inflammasomes act as an important part of the inflammatory process after SCI. Probenecid, an inhibitor of the Pannexin-1 channel, can inhibit the activation of inflammasomes. This article focuses on the effects of probenecid on the local immune microenvironment, histopathology, and behavior of SCI. Our data show that probenecid inhibited the expression and activation of nucleotide-binding oligomerization domain receptor pyrindomain-containing 1 (NLRP1), apoptosis-associated speck-like protein containing a CARD (ASC) and caspase-1, interleukin-1β (IL-1β), and caspase-3 proteins associated with inflammasomes, thereby suppressing the proportion of M1 cells. And consequently, probenecid reduced the lesion area and demyelination in SCI. Moreover, the drug increased the survival of motor neurons, which resulted in tissue repair and improved locomotor function in the injured SC. Altogether, existing studies indicated that probenecid can alleviate inflammation by blocking Pannexin-1 channels to inhibit the expression of caspase-1 and IL-1β, which in turn restores the balance of immune cell subsets and exerts neuroprotective effects in rats with SCI.

## Introduction

Spinal cord injury (SCI) is damage to the spinal cord caused by a sports accident, traffic accident, or fall from a height, which results in the loss of motor or sensory function, leading to a decrease in the quality of life and a heavy medical burden on the family and society (Gaojian et al., 2020). The treatment of SCI has been a challenge for the medical community because of the poor regenerative capacity of neurons after SCI and the rapid appearance of glial scarring, which makes it difficult to repair and reconstruct the spinal cord tissue and function (Wang et al., 2019). Therefore, it is significant to explore effective treatments for SCI to improve patients’ quality of life and reduce the medical burden for families and society.

Spinal cord injury contains two phases, the primary phase and the secondary phase (the main phase). The mechanisms of secondary injury after SCI are complex and include neuroinflammatory response, ischemia and hypoxia, lipid peroxidation, apoptosis, etc. (Wu and Xu, 2016). Among them, the neuroinflammatory response has been regarded as an important factor in the pathological process leading to secondary injury (Scholtes et al., 2012). The pathophysiological processes of secondary injury, such as apoptosis, edema, glial scarring, and inflammation in the injured tissue, severely impact the conduction function of nerves in the corresponding spinal cord segments. The inflammatory response during SCI includes local immune cell activation of the injury, the infiltration of peripheral immune cells, and the production of proinflammatory factors, which ultimately lead to spinal cord dysfunction (Sun et al., 2016). Effective and early use of anti-inflammatory drugs is of considerable importance to improve the local immune microenvironment and promote neuronal regeneration and recovery of spinal cord function.

Probenecid, a sulfonamide derivative approved for use in 1951, has been used to treat gout and its adverse effects, and pharmacokinetics have been intensively studied (Robbins et al., 2012). One study found that oxidative stress in osteoclast formation can be effectively inhibited by probenecid (Cheng and Kim, 2020). Another study showed that probenecid also acts as a pannexin-1 channel inhibitor and may inhibit the activation of inflammasomes (Silverman et al., 2009). Inflammasomes are multiprotein complexes that mainly consist of intracellular pattern recognition receptors (PRRs), apoptosis-associated speck-like protein containing a card (ASC), and pro-caspase-1 (Christgen et al., 2020). The pannexin-1 channel presents mainly in the brain, spinal cord, and thyroid gland (Bruzzone et al., 2003). It has a great effect on the activation of inflammasomes. In a study of a rat brain model of cognitive impairment, probenecid was found to inhibit the activation of nucleotide-binding oligomerization domain receptor pyrindomain-containing 1 (NLRP1) inflammasomes and reduce the activation of caspase-1, which in turn reduced the degree of cognitive impairment in rats (Mawhinney et al., 2011). Several studies have reported that probenecid can also reduce neuropathic pain in the spinal cord (Pineda-Farias et al., 2013; Bravo et al., 2014). These reports suggest that during the injury process of the central nervous system (CNS), probenecid has a neuroprotective and restorative effect on CNS injury, SCI has a similar process; however, it has not been reported whether this drug is useful for the treatment of SCI.

In the immune microenvironment at the site of SCI, there are generally M1 and M2 two phenotypes of macrophages. They appear in all stages from SCI to recovery, especially playing an indispensable part in the secondary inflammatory response of SCI (Martinez et al., 2008; Milich et al., 2019; Zhang et al., 2020). M1 cells can produce high levels of proinflammatory cytokines, such as secreting proinflammatory factors, like tumor necrosis factor α (TNF-α) and interleukin-1β (IL-1β), which induce the production of inflammatory cascade responses. M2 cells can secrete anti-inflammatory factors like IL-4, IL-10, and other factors, which reduce the inflammatory response and promote wound healing (Gensel and Zhang, 2015). Whether probenecid affects the polarization of microglia/macrophages to exert neuroprotective effects on SCI is an important research value to explore new mechanisms of SCI pathology and find new targets for treatment.

## Materials and Methods

### Experimental Animals

In this experiment, a total of 98 SPF-grade female Sprague Dawley (SD) rats (weight, 200–220 g; age, 8 weeks old) were used. All these rats were provided by Jinan Pengyue Experimental Animal Breeding Co., Ltd., Jinan, China, under license No. SCXK (Lu) 2019-0003. All surgical operations and postoperative care of SD rats during the experiment were approved by the Committee on the Laboratory Animal Care and Use of Bengbu Medical College.

### Experimental Methods

#### Preparation of Spinal Cord Injury Models in Adult Rats

Before performing the test, SD rats were housed in an environment with the appropriate humidity and temperature and given adequate feed and water for 5–7 days. Before surgery, the required surgical instruments were soaked in 75% alcohol (Shangdong Lierkang Co., Dezhou, China) for 30 min and sterilized with UV light for 30 min. During the operation, firstly, SD rats were anesthetized with 10% trichloroacetaldehyde intraperitoneally, then the rat hair around the T9 vertebral plate on the back of the rats was removed, the skin, fascia, and muscles of the corresponding part of T9 were cut open with a scalpel (Shanghai Medical Equipment Co., Shanghai, China), the T9 spinous process was removed with a bite forceps, and the vertebral plate was bitten along the interspinal foramen between T9 and T10 to fully expose the spinal cord, forming a circular opening of about 2.5 mm in diameter. Then, we use a 10-g weight falling directly from a certain height of about 25 mm to impact the exposed spinal cord. After surgery, the rats were grouped into different groups. For the sham-operated group [sham group], the rats received the same surgical procedure but without spinal cord damage and pharmacological treatment. SD rats after SCI were randomly divided into solvent control group [SCI (vector) group] (intraperitoneal injection within 3 h after surgery, followed by daily injection of PBS buffer (Biosharp) and the probenecid (Sigma) administration group [SCI (Prob) group; intraperitoneal injection of probenecid within 3 h after surgery, followed by daily injection (1 mg/kg)]. To prevent infection, the rats were given intraperitoneal injections of penicillin for 7 days. SD rats were given manual bladder emptying three times daily until the recovery of the bladder function.

#### Western Blot

Three days after SCI surgery, the rats were anesthetized with 10% trichloroacetaldehyde and then perfused with PBS, and total protein was extracted from the spinal cord tissue of the injury site (0.5-cm spinal cord segments containing the injury epicenter and the same segments for the sham group) and treated with RIPA lysis (Beyotime). We use the BCA kit (CWBIO) to detect the protein concentration and then use the SDS-PAGE gel kit (Beyotime) with the corresponding concentration to separate the protein, and then transfer it to polyvinylidene fluoride (PVDF) membranes (Millipore). The PVDF membranes were blocked with 5% skim milk for 2 h, then the primary antibodies incubated PVDF membranes overnight at 4°C. After that, the PVDF membranes were incubated for 2 h with horseradish peroxidase- (HRP-) conjugated secondary antibody. Proteins were detected with Immobilon western chemilum HRP substrate (Millipore) and observed with an electrochemiluminescence gel imager (Bio-Rad). Primary and secondary antibody information is listed in Supplementary Table 1. Finally, the expression of each protein was quantified using Image J.

#### Immunofluorescence Staining

At 7 days following surgery, the rats were anesthetized with 10% trichloroacetaldehyde, as described in the western blot protocol. And, the rats were cardiac perfused with PBS followed by 4% pre-cooled paraformaldehyde (PFA) at 4°C until the rats’ limbs were rigid, 1-cm spinal cord segments containing the injury epicenter were removed and postfixed overnight in 4% PFA before being transferred to 30% sucrose in 0.01 M PBS (pH 7.4) at 4°C overnight. Spinal cords were embedded in an OCT compound embedding medium (TissueTek, Miles, Elkart) and 10-μm serial frozen sections were prepared using a frozen microtome (Leica), followed by thaw-mounting on poly-L-lysine-coated slides. For the immunohistochemical assay, firstly, the tissue sections were incubated with the primary antibody overnight at 4°C. After that, the sections were washed with PBS and then incubated with the secondary antibody for 2 h. Next, the tissue sections were washed with PBS, and then we used blue nuclear dye (Hoechst 33342; Abcam) to stain the cell nuclei, and the tissue sections were covered with coverslips for use. Supplementary Table 2 shows the details of the antibodies. Photographs were taken with a fluorescent microscope (Zeiss). In the quantitative experiment of immunofluorescence, the spinal cord cross-sections of six rats were used for evaluation. We took multiple pictures around the center of the SCI. Double-positive cells in each picture were counted by the blinded observer, and the average value was divided by the picture area. A smaller area in each group of pictures was selected as the representative picture.

#### Flow Cytometry

Rats were anesthetized with 10% trichloroacetaldehyde and then perfused with PBS after 7 days of SCI. Then, 0.5-cm spinal cords, including the injury epicenter or the same segments for the sham group, were removed. The spinal cords were then grounded and centrifuged to obtain a single-cell suspension. Then, mononuclear cells were isolated by Percoll (Solarbio) gradient centrifugation. Briefly, 70% Percoll solution, 30% Percoll solution, and cell suspensions were added to a 15 ml conical tube in sequence, the cells were centrifuged at 300 g for 30 min at 20°C. After centrifugation, the cell debris layer was discarded and the rest parts were washed two times before their use. Then, the primary antibodies were incubated with the cells for 30 min; after that, the cells were washed with PBS and then fixed with 0.5 ml of 1% PFA (MACKLIN); finally, they were detected by BD FACSVerse flow cytometer (BD Bioscience). We used isotype control antibodies to estimate the non-specific staining that was subtracted from the specific staining results. Supplementary Table 3 shows the details of the antibodies. We use FlowJo 7.6 software (FlowJo) to analyze the data.

#### Histological Analysis

The remaining rats were anesthetized with 10% trichloroacetaldehyde at 6 weeks post-SCI, and cardiac perfusion was performed with PBS followed by precooled PFA at 4°C. Spinal cords were collected, fixed, and cut into 10 μm serial frozen sections using a frozen microtome (Leica), as described in the immunofluorescence protocol. According to the manufacturer’s instructions, the slides were stained with the desired staining. Hematoxylin-eosin (HE, Beyotime) and Luxol Fast Blue (LFB, Sigma-Aldrich) staining were performed to measure the lesion area and the preservation of myelination (LFB-positive area) of the spinal cord. Lesion and myelinated area measurements were performed in an unbiased stereological manner using ImageJ software. Cavitation and LFB-positive tissue at the injury epicenter and the epicenter to rostral and caudal 1, 2, 3, and 4 mm in axial sections were quantified and normalized to the percentage of intact spinal cord area. Nissl staining (Beyotime) was used to identify surviving motor neurons by the existence of the Nissl substance and euchromatic nuclei. Surviving motor neurons were quantified by counting all such cells in the ventral horn. The three staining methods were described previously (Chen et al., 2020).

#### Behavioral Analysis

Three methods were used to assess the behavior of SCI rats. The Basso, Beattie, and Bresnahan (BBB) locomotor rating scale ranging from 0 to 21 points was used to assess the behavior of SCI rats. The BBB locomotor rating scale was performed by the double-blind method at 1, 3, 5, 7, 14, 28, 35, and 42 days after SCI, when the rats walked freely on the open-field surface for 4 min. The footprint analysis and the Grid walk test were performed as previously described (Metz et al., 2000). Information from the BBB locomotor rating scale and footprint analysis is listed in Supplementary Tables 4 and 5.

#### Statistical Analyses

Data are presented as mean ± SD. Histological data were analyzed by a two-way ANOVA followed by Bonferroni’s multiple comparisons test, and the BBB locomotor rating scale was analyzed by repeated-measures two-way ANOVA followed by Tukey’s multiple comparisons test. A one-way ANOVA with a Tukey’s multiple comparisons test and student’s *t*-tests were used to evaluate the other data. *p* < 0.05 was considered statistically significant. Data were analyzed with Graphpad Prism software v.9.0.

## Results

### Probenecid Inhibits the Expression and Activation of Inflammasome-Associated Molecules After Spinal Cord Injury

**FIGURE 1:**
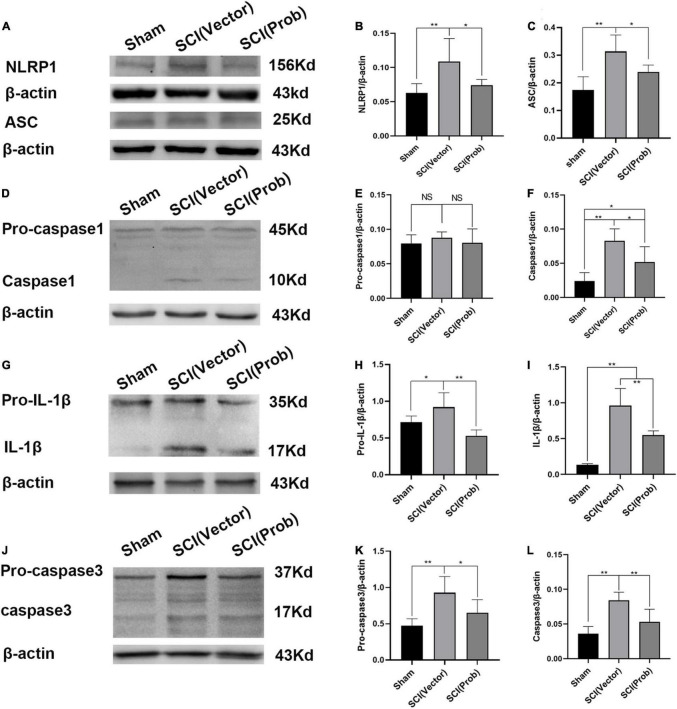
Probenecid inhibits the expression of inflammasome-associated molecules at 3 days following spinal cord injury (SCI). **(A,D,G,J)** Representative pictures of WB results of the expression of NLRP1 and ASC **(A)**, pro-caspase-1and caspase-1 **(D)**, pro-interleukin-1β (pro-IL-1β) and IL-1β **(G)**, pro-caspase-3 and caspase-3 **(J)**. **(B,C,E,F,H,I,K,L)** Quantitative results of the expression of inflammasome-associated molecules (*p < 0.05, **p < 0.01 *n* = 6).

To prove the effect of probenecid on the expression and activation of inflammasome-associated molecules, we performed western blotting on spinal cord homogenate extracts obtained from sham, SCI (vector), and SCI (Prob) groups. ASC and NLRP1 were significantly increased in the SCI (vector) group, whereas they were significantly reduced in the SCI (Prob) group compared with the SCI (vector) group (Figures 1A–C). Pro-caspase-1 levels were unchanged among the three groups (Figures 1D–F). However, caspase-1 levels were significantly decreased in the SCI (Prob) group compared with the SCI (vector) group. Pro-IL-1β and IL-1β levels were significantly decreased after probenecid treatment compared with the SCI (vector) group (Figures 1G–I). The levels of the pro-caspase-3 and the caspase-3 were significantly decreased in the SCI (Prob) group compared with the SCI (vector) group (Figures 1J–L).

**FIGURE 2:**
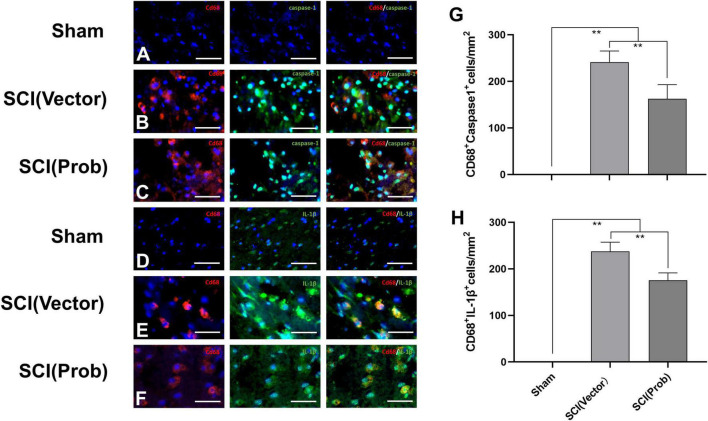
Inhibitory effects of Probenecid on the expression of inflammasome-associated molecules at 7 days following SCI: immunofluorescence detection. Representative images of CD68 (red, rhodamine staining) and caspase-1 [green, fluorescein isothiocyanate (FITC) staining] co-stained **(A–C)**, CD68 and IL-1β (green, FITC staining) co-stained **(D–F)**. Hoechst 33342 (blue) was used to counterstain cells to visualize nuclei. **(G,H)** Quantitative analysis of CD68^+^caspase-1^+^ **(G)**, and CD68^+^ IL-1β^+^ **(H)**, (**p < 0.01 *n* = 6) scale bar: 50 μm.

The immunofluorescence assay was used to detect the effect of probenecid on the expression and activation of inflammasome-associated molecules. We used double immunofluorescence staining to label CD68 with caspase-1 and IL-1β, respectively (Figures 2A–F). CD68 is the marker of activated microglia/macrophages. The number of CD68^+^ Caspase-1^+^ cells and CD68^+^ IL-1β^+^ cells was significantly decreased in the SCI (Prob) group compared with the SCI (vector) group (Figures 2G,H). The results could indicate that probenecid inhibits the expression and activation of inflammasome-associated molecules after SCI.

### Probenecid Inhibits Spinal Cord Injury-Induced Polarization of Microglia/macrophages Into M1 Cells and Increases the Polarization of Microglia/macrophages Into M2 Cells

**FIGURE 3:**
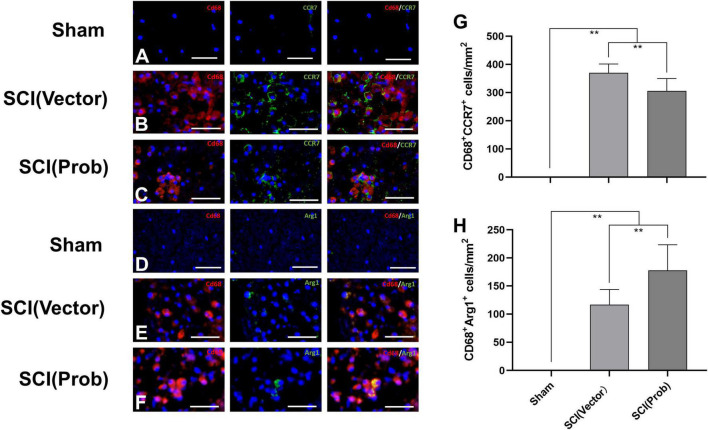
Effects of Probenecid on M1 and M2 cells in the injured spinal cord as distinguished by immunofluorescence. Representative images of CD68 (red, rhodamine staining) and CCR7 (green, FITC staining) co-stained M1 cells **(A–C)**, CD68 (red, rhodamine staining) and Arg1 (green, FITC staining) co-stained M2 cells **(D–F)**. Cells were counterstained with Hoechst 33342 (blue) to visualize nuclei. **(G,H)** Quantitative analysis of CD68^+^ CCR7^+^ **(G)**, and CD68^+^ Arg1^+^ (**H**; **p < 0.01 *n* = 6), scale bar: 50 μm.

To verify the effect of probenecid on M1 and M2 phenotype cells, we used the immunofluorescence assay to detect the specific markers of M1 (CD68^+^CCR7^+^) and M2 cells (CD68^+^Arg1^+^; Figures 3A–F). The number of CD68^+^CCR7^+^ (M1) cells was significantly decreased and the number of CD68^+^Arg1^+^ (M2) cells was significantly increased in the SCI (Prob) group compared with the SCI (vector) group (Figures 3G,H).

**FIGURE 4:**
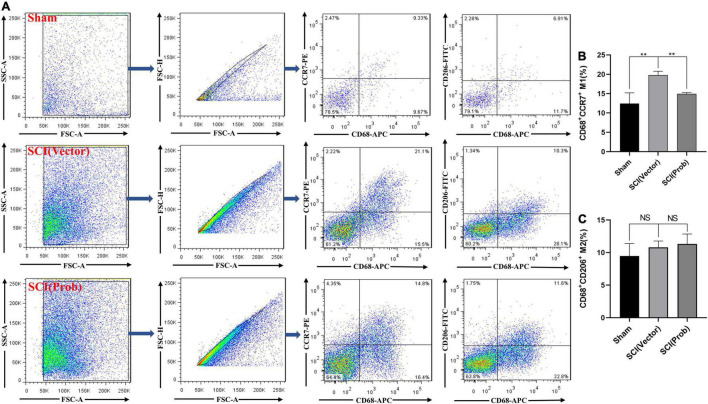
Effects of Probenecid on Polarization of M1 and M2 in the injured spinal cord as distinguished by flow cytometr (FCM). CD68^+^CCR7^+^ and CD68^+^CD206^+^ cells were defined as M1 and M2, respectively. **(A)** Flow cytometry pictures of cells derived from spinal cord homogenate. **(B,C)** Statistical charts (**p < 0.01 *n* = 4).

M1 and M2 cells in the injured spinal cords were also analyzed by flow cytometry. CD68^+^CCR7^+^ and CD68^+^CD206^+^ cells were defined as M1 and M2 cells, respectively (Figure 4A). Although the proportions of M2 cells did not differ between the SCI (Prob) and SCI (vector) group (Figure 4C), the proportions of M1 cells were significantly increased in the SCI (vector) compared with the sham group while the proportions of M1 cells after probenecid treatment were significantly decreased compared with the SCI (vector) group (Figure 4B). This is enough to indicate that probenecid can improve the local immune microenvironment of SCI.

### Probenecid Reduces Spinal Cord Tissue Damage, Increases Myelination and the Number of Residual Neurons, and Promotes Functional Recovery

**FIGURE 5:**
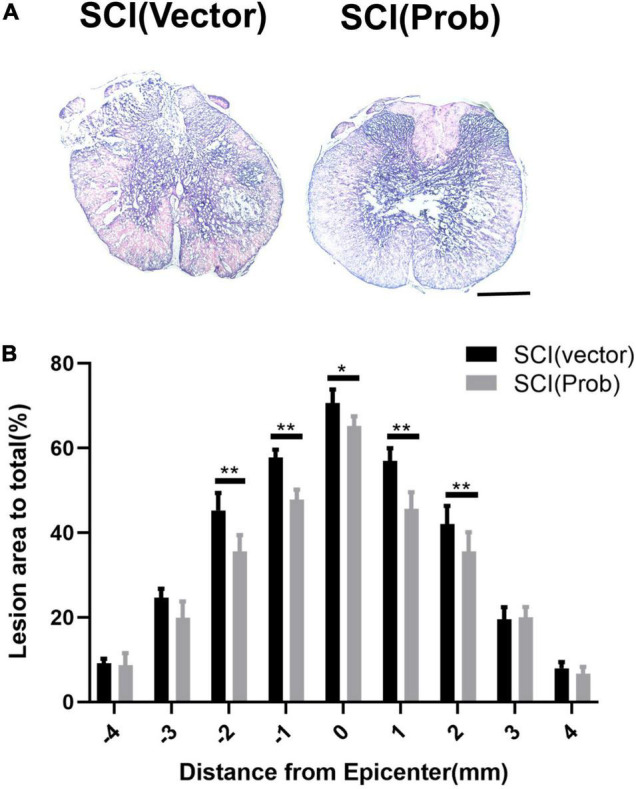
Quantitative analysis of the effect of Probenecid on lesion areas. **(A)** Representative pictures of hematoxylin-eosin (HE) staining in the transverse sections of 2 mm rostral to the epicenter of spinal cords. **(B)** Quantitative analysis of lesion areas in different groups at different distances from the epicenter (0 mm) and 1, 2, 3, and 4 mm rostral (+) and caudal (–) from the epicenter (*p < 0.05, **p < 0.01 *n* = 6). Scale bar: 500 μm.

**FIGURE 6:**
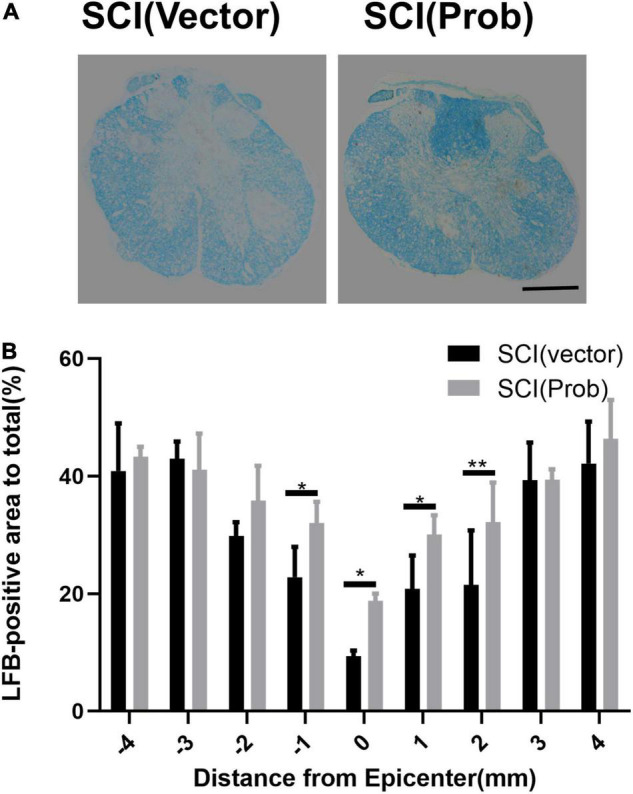
Quantitative analysis of the effect of Probenecid on myelin preservation. **(A)** Representative pictures of LFB staining in the transverse sections of 2 mm rostral to the epicenter of spinal cords. **(B)** Quantitative analysis of residual myelination in different groups at different distances from the epicenter (0 mm) and 1, 2, 3, and 4 mm rostral (+) and caudal (–) from the epicenter (*p < 0.05, **p < 0.01 *n* = 6). Scale bar: 500 μm.

**FIGURE 7:**
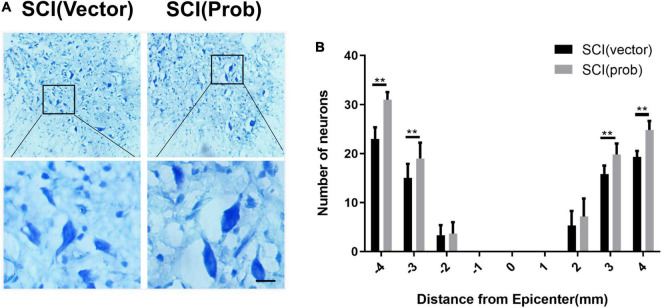
Quantitative analysis of the effect of Probenecid on the number of residual ventral horn neurons. **(A)** Nissl-stained images of neurons in the ventral horn 3 mm rostral to the epicenter at 6 weeks post-SCI. Scale bar: 50 μm. **(B)** Quantitative analysis of residual ventral horn neurons in different groups at different distances from the epicenter of the injury (0 mm) and 1, 2, 3, and 4 mm rostral (+) and caudal (–) from the epicenter. (**p < 0.01 *n* = 6).

To verify the effect of probenecid on histopathology and behavior after SCI, myelin preservation, the area of lesion, and motor neuron survival were examined by LFB, HE, and Nissl staining, respectively. Figures 5A, 6A are the representative pictures of HE and LFB staining in the 2-mm transverse sections rostral to the epicenter of spinal cords. Lesion areas in the center of injury, 1 and 2 mm rostral and caudal to the epicenter were smaller in the SCI (Prob) group than in the SCI (vector) group (Figure 5B). The LFB-positive areas at the lesion epicenter, 1 mm rostral and caudal, and 2 mm rostral to the epicenter in the SCI (Prob) group were larger than those in the SCI (vector) group (Figure 6B). Figure 7A is a representative Nissl-staining image of neurons in the ventral horn 3 mm rostral to the lesion at 6 weeks post SCI. The number of residual ventral horn motoneurons 3 and 4 mm rostral and caudal to the injury center in the SCI (Prob) group were more than those in the SCI (vector) group (Figure 7B).

**FIGURE 8:**
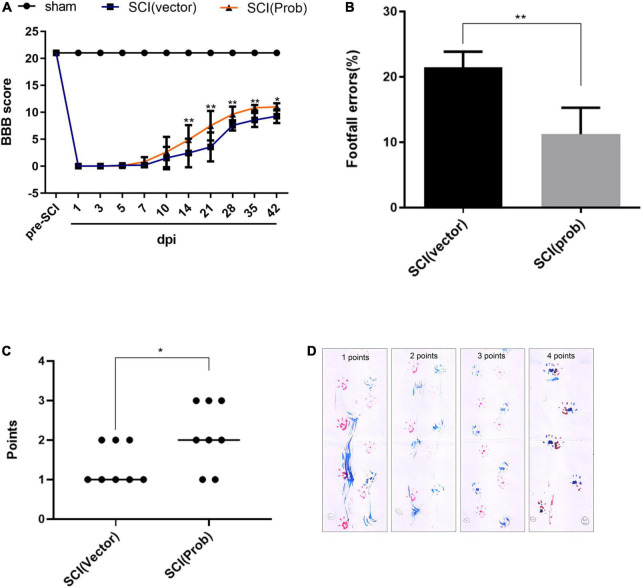
Effects of Probenecid on behavior recovery following SCI. Motor function was evaluated by the BBB scoring, footprint analysis, and grid walk in the SCI (Prob) and SCI (vector) group. **(A)** The BBB scores of the three groups. **(B)** Grid walk test post SCI. **(C)** Footprint analysis post SCI. **(D)** Representative pictures of footprints of different points (*p < 0.05, **p < 0.01 *n* = 8).

To research the effect of probenecid on behavioral recovery after SCI, BBB scores were performed at 1, 3, 5, 7, 10, 14, 21, 28, 35, and 42 days after SCI. Both the sham group and the pre-injury score were 21, and there was no significant difference between the SCI (Prob) group and the SCI (vector) group from 1 to 10 days post-injury. At 14, 28, 35, and 42 days, scores were higher in the SCI (Prob) group than in the SCI (vector) group (Figure 8A). At 6 weeks following SCI, a grid walk test and the footprint analysis were also used to evaluate the effects of neuroprotection and motor function recovery. The SCI (Prob) group had less footfall errors compared to the SCI (vector) group (Figure 8B). Similarly, in the footprint analysis, the scores of the SCI (vector) group were lower than those of the SCI (Prob) group (Figures 8C,D).

## Discussion

Spinal cord injury is a direct or indirect injury to the spinal cord, which has a high incidence and disability rate, causing pain and suffering to the patient and a heavy burden to the family and society. The pathogenesis of SCI is complex and has not yet been precisely elucidated. It has been reported that a series of cascade reactions, including neuroinflammation, apoptosis, and free radical production, occur during the development of SCI (Rong et al., 2019; Zhu et al., 2020). In this experiment, we establish SCI models in SD rats to investigate the function of probenecid in SCI and its possible mechanism.

Probenecid is a compound with a molecular weight of 285.36 Da, and its molecular formula is C_13_H_19_NO_4_S, which has been used for the treatment of gout clinically (Robbins et al., 2012). Probenecid has been found to inhibit oxidative stress in neuronal cells (Cheng and Kim, 2020) and also acts as a pannexin-1 channel inhibitor, which may inhibit the activation of inflammasomes (Silverman et al., 2009), as well as being protective in a rat model of cognitive impairment (Mawhinney et al., 2011). These reports suggest that probenecid is therapeutic for CNS injury. Recently, we also found that some signal pathways associated with inflammatory responses can be inhibited by the application of probenecid after SCI *via* the RNA-sequencing analysis (Zhang et al., 2020). Therefore, we hypothesize that probenecid may have anti-inflammatory and neuroprotective effects. The pannexin-1 channel is mainly found in the brain, spinal cord, and thyroid and is the most widely studied channel due to its key role in the activation of inflammasomes (Bruzzone et al., 2003). The literature suggested that it was involved in many diseases, such as Alzheimer’s disease, type II diabetes, and atherosclerosis (Karasawa and Takahashi, 2017; Sepehri et al., 2017; White et al., 2017). Some of the inflammasomes involved in pannexin-1 channels, like NLRP1, NLRP2, and NLRP3, have been shown to exert effects in inflammation due to CNS injury (de Rivero Vaccari et al., 2014; Mortezaee et al., 2018). During the process of natural immune defense, the activation of caspase-1 can be regulated by inflammasomes and foster the creation of IL-1β and IL-18 (Lamkanfi and Dixit, 2012; Strowig et al., 2012), ultimately leading to the generation of a series of downstream inflammatory cascade responses. We hypothesized that the pannexin-1 channel could act as an important part to inhibit the activation of inflammasomes, thereby improving the immune microenvironment of SCI and reducing spinal cord nerve injury. It has been shown that probenecid can treat neuropathic pain due to its inhibition of pannexin-1 channels (Bravo et al., 2014). Our experimental results showed that probenecid inhibits the expression and activation of pannexin-1 channel-related molecules, like ASC, NLRP1, IL-1β, and caspase-1 after SCI. The results indicated that probenecid can affect the expression of inflammatory factors downstream of inflammasomes by inhibiting pannexin-1 channels in SCI rat models. Following SCI, inflammatory cells at the site of injury are activated and increased, producing large amounts of inflammatory factors and forming an inflammatory microenvironment, eventually resulting in spinal cord dysfunction (Sun et al., 2016). It has been proven that, under natural conditions, inflammatory cell subsets (e.g., M1) constitute the majority in the local immune microenvironment of SCI, while anti-inflammatory cell subsets (e.g., M2) are fewer (Zhou et al., 2014; Ma et al., 2015; Ahmed et al., 2018), which is an important mechanism for the pathological damage that occurs after SCI. As probenecid inhibits pannexin-1 channels, suppresses the stimulation of inflammasomes, and reduces proinflammatory factors, we hypothesize that probenecid may improve the local immune microenvironment and exert effects to protect the nerve. We used flow cytometry to investigate the effects of probenecid on local immune cell subsets in SCI by detecting CD68, a universal marker of activated microglia/macrophages, and specific markers of M1 (CCR7^+^) and M2 subtype cells (CD206^+^). The results showed a significant increase in M1-type macrophages after SCI, which is consistent with the pattern that M1-type macrophages promote the development of inflammatory responses. In the SCI (Prob) group, we found that there was a decrease in M1 cells, which may be the result of prohibiting inflammasome activation by probenecid. We also used the immunofluorescence assay to investigate the effects of probenecid on local immune cell subsets by detecting the specific markers of M1 (CCR-7) and M2 cells (Arg1). Our study indicated that probenecid lessened the number of M1 cells and rose the number of M2 cells compared with the SCI (vector) group. Therefore, we hypothesize that probenecid could affect the polarization of microglia/macrophages, thereby affecting the local immune microenvironment.

These findings suggested that the inhibition of inflammasome activation might reduce M1 cells and increase M2 cells (Ślusarczyk et al., 2018; Su et al., 2020). Taken together, probenecid could inhibit the activation of inflammasomes, thereby affecting microglia/macrophage cell polarization to improve the limited immune microenvironment. These findings supported the possibility that probenecid may provide neuroprotection and improve motor function. Morphological and behavioral tests were also performed to determine whether probenecid has such effects. In the SCI (Prob) group, a meaningful decline in the demyelinating area of spinal cord, a rise in residual ventral horn motor neurons and a considerable improvement in motor function were observed.

## Conclusion

In summary, the present experimental results indicate that probenecid is restorative in the SCI model in SD rats. The possible mechanism is that probenecid improves rat SCI by inhibiting the activation of inflammasomes, reducing the production of proinflammatory factors, improving the local immune microenvironment, reducing the early inflammatory response, and reducing secondary SCI through the inhibition of Pannexin-1 channels, and the early use of probenecid may be a meaningful strategy for the treatment of SCI.

## Data Availability Statement

The raw data supporting the conclusions of this article will be made available by the authors, without undue reservation.

## Ethics Statement

The animal study was reviewed and approved by Committee on the Laboratory Animal Care and Use of the Bengbu Medical College.

## Author Contributions

Y-XZ participated in literature search, study design, and writing. H-ZL participated in the study design and data interpretation. QQ and X-XW performed experimental procedures and statistically analyzed the data. J-LL, Y-QC, J-RC, and JX conducted data analysis and figures. All authors read and approved the final manuscript.

## Conflict of Interest

The authors declare that the research was conducted in the absence of any commercial or financial relationships that could be construed as a potential conflict of interest.

## Publisher’s Note

All claims expressed in this article are solely those of the authors and do not necessarily represent those of their affiliated organizations, or those of the publisher, the editors and the reviewers. Any product that may be evaluated in this article, or claim that may be made by its manufacturer, is not guaranteed or endorsed by the publisher.
