# Correction: Neuroprotective effects of the pannexin-1 channel inhibitor: probenecid on spinal cord injury in rats

**DOI:** 10.3389/fnmol.2025.1698001

**Published:** 2025-09-26

**Authors:** Qi Qi, Xiao-Xuan Wang, Jing-Lu Li, Yu-Qing Chen, Jian-Rong Chang, Jin Xi, He-Zuo Lü, Yu-Xin Zhang

**Affiliations:** ^1^Clinical Laboratory, The First Affiliated Hospital of Bengbu Medical College, Bengbu, China; ^2^Anhui Key Laboratory of Tissue Transplantation, Bengbu Medical College, Bengbu, China; ^3^School of Laboratory Medicine, Bengbu Medical College, Bengbu, China; ^4^School of Basic Medicine, Bengbu Medical College, Bengbu, China

**Keywords:** probenecid, spinal cord injury, inflammasome, macrophages polarization, immune microenvironment

In the published article, the reference for “Baranova et al., 2004; Papadopoulos and Verkman, 2008” was erroneously written as “Baranova, A., Ivanov, D., Petrash, N., Pestova, A., Skoblov, M., Kelmanson, I., et al. (2004). The mammalian pannexin family is homologous to the invertebrate innexin gap junction proteins. Genomics 83, 706–716. doi: 10.1016/j.ygeno.2003.09.025; Papadopoulos, M. C., and Verkman, A. S. (2008). Potential utility of aquaporin modulators for therapy of brain disorders. Prog. Brain Res. 170, 589–601. doi: 10.1016/S0079-6123(08)00446-9”. It should have instead been correctly written as “Robbins, N., Koch, S.E., Tranter, M., and Rubinstein, J. (2012). The history and future of probenecid. *Cardiovasc. Toxicol*. 12, 1–9. doi: 10.1007/s12012-011-9145-8”.

In the published article, the reference for “Cheng and Kim, 2020” in the **Discussion** section, Paragraph 2 was erroneously written as “Cheng, M. H., and Kim, S. J. (2020). Inhibitory effect of probenecid on osteoclast formation via JNK, ROS and COX-2. Biomol. Ther. (Seoul.) 28, 104–109. doi: 10.4062/biomolther.2019.047”. It should have instead been correctly written as “Robbins, N., Koch, S.E., Tranter, M., and Rubinstein, J. (2012). The history and future of probenecid. *Cardiovasc. Toxicol*. 12, 1–9. doi: 10.1007/s12012-011-9145-8”.

In the published article, the reference for “Cheng and Kim, 2020” in the **Discussion**, Paragraph 2 was erroneously written as “Cheng, M. H., and Kim, S. J. (2020). Inhibitory effect of probenecid on osteoclast formation via JNK, ROS and COX-2. Biomol. Ther. (Seoul.) 28, 104–109. doi: 10.4062/biomolther.2019.047”. It should have instead been correctly written as “Mawhinney, L. J., de Rivero Vaccari, J. P., Dale, G. A., Keane, R. W., and Bramlett, H. M. (2011). Heightened inflammasome activation is linked to age-related cognitive impairment in Fischer 344 rats. *BMC Neurosci*. 12:123. doi: 10.1186/1471-2202-12-123”.

In the published article, in the **Materials and Methods** section, *Experimental Animals*, paragraph 1, the phrase “women standard deviation (SD) rats” was erroneously included. This has now been corrected to: “female Sprague Dawley (SD) rats”.

In the published article, in both the **Introduction**, paragraph 3, and the **Discussion section**, paragraph two, the phrase “nerve cells” was erroneously included. This has now been corrected to: “osteoclast formation”.

The original version of this article has been updated.

