# Supplementary material for: Neuroprotective Effects of the Pannexin-1 Channel Inhibitor: Probenecid on Spinal Cord Injury in Rats
Source: Front Mol Neurosci. 2022 May 19;15:848185. doi: 10.3389/fnmol.2022.848185 (PMC9162172; doi:10.3389/fnmol.2022.848185)
Supplement: Supplementary file 1 [file Table_1.docx]

***Supplementary information***

| ***Table 1.* *Information of antibodies used in Western blot*** | | | | |
| --- | --- | --- | --- | --- |
| Antigen | Dilution | Company | Host species | Catalogue # |
| ASC | 1:1000 | Affinity | Rabbit | DF6304 |
| NLRP1 | 1:1000 | Abcam | Rabbit | ab3683 |
| Caspase3 | 1:1000 | Abcam | Rabbit | Ab13847 |
| IL-1β | 1:1000 | Abcam | Rabbit | ab9722 |
| Caspase1 | 1:1000 | Abcam | Rabbit | Ab179515 |
| β-actin | 1:1000 | Biosharp | Rabbit | BL005B |
| Rabbit IgG (H + L) | 1:10000 | Biosharp | Goat | BL003A |

***Table 2. Information of antibodies used in Immunofluorescence staining***

| Antigen | Host species | Cat. # or Lot # | RRID | Company |
| --- | --- | --- | --- | --- |
| CD68 | Mouse | MA513324 | AB_10987212 | Invitrogen |
| CCR7 | Rabbit | ab191575 |  | Abcam |
| ARG1 | Rabbit | PA5-29645 | AB_2547120 | Invitrogen |
| Caspase1 | Rabbit | 22915-i-ap |  | Proteintech |
| IL-1β | Rabbit | Ab9722 |  | Abcam |

***Table 3.* *Information of antibodies used in Flow cytometry***

| Antigen | Conjugation | Company | Host species | Catalogue # |
| --- | --- | --- | --- | --- |
| CD68 | Alexa Flour 647 | BIO-RAD | Mouse | MCA341A 647 |
| IgG1 isotype control | Alexa Flour 647 | CST | Mouse | 4843S |
| CCR7 | PE | BIOSS | Rabbit | BS-1305r-PE |
| IgG isotype control | PE | BIOSS | Rabbit | BS-0295p- PE |
| CD206 | FITC | BIOSS | Rabbit | BS-4727r-FITC |
| IgG isotype control | FITC | BIOSS | Rabbit | BS-0295p-FITC |

***Table 4.* *Information of*** ***BBB locomotor rating scale***

| Score | Criteria | |
| --- | --- | --- |
| 0 | No observed movement of the hindlimbs | |
| 1  2 | Slight movement of one or two joints,usually hip or knee  Extensive movement of one joint or extensive movement of one joint and c movement of the other. | |
| 3 | Extensive movement of two joints. | |
| 4 | Slight movement of all three joints of the hindlimbs. | |
| 5 | Slight movement of two joints and extensive movement of the third joint. | |
| 6  7  8 | Extensive movement of two joints and slight movement of the third joint.  Extensive movement of the three joints in the hindlimbs.  Sweeping without weight bearing or plantar support of the paw without weight bearing | |
| 9  10    11  12  13  14  15  16  17  18  19  20  21 | Plantar support of the paw with weight bearing only in the support stage or occasional,frequent or inconsistent dorsal stepping with weight bearing and no  plantar stepping.  Plantar stepping with occasional weight bearing and no forelimb-hindlimb coordination.  Plantar stepping with frequent to consistent weight bearing and no forelimb-hindlimb coordination.  Plantar stepping with frequent to consistent weight bearing and occasional  forelimb-hindlimb coordination.  Plantar stepping with frequent to consistent weight bearing and frequent forelimb-hindlimb coordination.  Plantar stepping with consistent weight support,consistent forelimb-hindlimb coordination and predominnatly rotated paw position during locomotion both at the instant of initial contact with the surface as well as before moving the toes at the end of the support stage or frequent plantar stepping, consistent forelimb-hindlimb coordination and occasional dorsal stepping.  Consistent plantar stepping, consistent forelimb-hindlimb coordination and no movement of the toes or occasional movement during forward movement of limb; predominant paw position is parallel to the body at the time of initial contact.  Consistent plantar stepping and forelimb-hindlimb coordination during gait and movement of the toes occurs frequently during forward movement of the limb;the predominant paw position is parallel to the body at the time of initial contact and curved at the instant of movement.  Consistent plantar stepping and forelimb-hindlimb coordination during gait and movement of the toes occurs frequently during forward movement of the limb;the predominant paw position is parallel to the body at the time of initial contact and at the instant of movement of the toes.  Consistent plantar stepping and forelimb-hindlimb coordination during gait and movement of the toes occurs consistently during forward movement of the limb;the predominant paw position is parallel to the body at the time of initial contact and curved during movement of the toes.  Consistent plantar stepping and forelimb-hindlimb coordination during gait and movement of the toes occurs consistently during forward movement of the limb;the predominant paw position is parallel to the body at the instant of contact and at the time of movement of the toes, and the animal presents a downward tail some or all of the time.  Consistent plantar stepping and forelimb-hindlimb coordination during gait and movement of the toes occurs consistently during forward movement of the limb;the predominant paw position is parallel to the body at the instant of contact and at the time of movement of the toes, and the animal presents consistent elevation of the tail and trunk instability.  Consistent planter stepping and coordinated gait;consistent movement of the toes; paw position is predominantly parallel to the body during the whole support stage; consistent trunk stability; consistent tail elevation. |  |

***Table 5.* *Information of*** ***Footprint analysis***

| Score | Criteria |
| --- | --- |
| 0 | Completely dragged |
| 1 | At least three consecutive footprints |
| 2 | No dragging at all |
| 3 | Mostly the same as 2, with the difference that the hindlimb landing point is parallel to the forelimb landing point |
| 4 | Normal rats |

***Table 6. List of abbreviations***

| ***Abbreviations*** | ***Full name*** |
| --- | --- |
| SCI | Spinal Cord Injury |
| ASC | Apoptosis-associated speck-like protein containing a card |
| CNS | Central nervous system |
| SD  TBS  PBS  PVDF  SDS-PAGE  RIPA  PMSF  PFA  BCA | Sprague Dawley  Tris-Hcl buffer solution  Phosphate buffer solution  Polyvinylidene fluoride  Sodium dodecyl sulphate-polyacrylamide gel electrophoresis  Radio-Immunoprecipitation Assay  Phenylmethanesulfonyl fluoride  Paraformaldehyde  Bicinchoninicacid |
